# Can neoadjuvant chemoradiotherapy combined with immunotherapy benefit patients with microsatellite stable locally advanced rectal cancer? a pooled and integration analysis

**DOI:** 10.3389/fonc.2023.1280995

**Published:** 2023-10-06

**Authors:** Yumin Yue, Min Cheng, Xiaohui Xi, Quan Wang, Mingtian Wei, Bobo Zheng

**Affiliations:** ^1^ Department of General Surgery, Shaanxi Provincial People’s Hospital, Xi’an, China; ^2^ Department of Colorectal Surgery, Xi'an International Medical Center Hospital, Xi’an, Shaanxi, China; ^3^ Ambulatory Surgery Center of Xijing Hospital, Fourth Military Medical University, Xi’an, China; ^4^ Department of Gastrointestinal Surgery, West China Hospital, Sichuan University, Chengdu, China

**Keywords:** neoadjuvant chemoradiotherapy, immunotherapy, microsatellite stable, rectal cancer, pooled and integration analysis

## Abstract

**Objective:**

To assess the clinical efficacy of neoadjuvant chemoradiotherapy combined with immunotherapy for patients with microsatellite stable (MSS) locally advanced rectal cancer and provide evidence to support clinical decision-making.

**Methods:**

A systematic search was conducted on the PubMed, Embase, Cochrane Collaboration databases, conference summaries, and Chinese databases for clinical studies that investigated neoadjuvant chemoradiotherapy combined with immunotherapy for the treatment of locally advanced rectal cancer with MSS status. The search spanned from the inception of each database through July 2023. Data from the identified studies were extracted using a pre-designed table, and efficacy outcomes were analyzed. An integrated analysis was conducted using Stata 12.0 software.

**Results:**

Eight studies were included, comprising 204 patients with locally advanced MSS rectal cancer who received chemoradiotherapy combined with immunotherapy. The integrated analysis revealed a pathologic complete remission rate of 0.33, a sphincter preservation rate of 0.86, an R0 resection rate of 0.83, a major pathologic remission rate of 0.33, and a clinical complete remission rate of 0.30.

**Conclusion:**

Neoadjuvant chemoradiotherapy combined with immunotherapy demonstrates significant short-term efficacy in MSS-type locally advanced rectal cancer, notably enhancing the pathologic complete remission and sphincter preservation rates. This combination is a recommended treatment for patients with MSS-type rectal cancer.

## 1 Introduction

Neoadjuvant chemoradiotherapy, when combined with surgery and followed by adjuvant chemotherapy, remains a primary treatment strategy for stage II/III locally advanced rectal cancer (LARC). Research indicates that after neoadjuvant radiotherapy, the local recurrence rate for advanced rectal cancer is maintained between 5% and 7% (1–3). A phase II clinical trial verified that six cycles of mFOLFOX6 after chemoradiotherapy and total mesorectal excision significantly elevated the pathologic complete remission (PCR) rate (4). Despite these advances, enhancing the PCR rate and managing distant metastasis remain central challenges. Emphasizing improved clinical outcomes, treatments for LARC now prioritize both survival duration and quality of life. Achieving complete tumor remission, especially for low rectal cancer patients, can lead to anus preservation, which holds immense clinical importance.

Recent discoveries highlight the efficacy of immune checkpoint inhibitors of PD1 in the advanced treatment of various tumors (5). While immunotherapy is particularly effective for colorectal cancer patients with mismatch repair gene expression deficiency or those with high microsatellite instability (MSI-H, which is associated with higher tumor mutation load and tumor-infiltrating lymphocytes [TILs]), Several studies have shown that the microsatellite instability status of tumors is an independent indicator of survival and prognosis of colorectal cancer patients with prognostic significance. Patients with MMR-deficient colorectal cancer generally have a better prognosis than patients with non-MMR-deficient cancer (6, 7). Therefore, the microsatellite instability status of tumors can be an essential influence on the prognosis of colorectal cancer patients, prompting physicians to quantify treatment and long-term follow-up. It has been shown that microsatellite instability can enhance tumor immunogenicity to a certain extent, as well as recognize and kill tumor cells through a variety of immune cells. Therefore, microsatellite instability may be an adjunctive indicator in immunotherapy (8, 9). However, 95% of rectal cancer is of the microsatellite stable (MSS) type. This type typically does not respond well to standalone immunotherapy (10). Many malignant tumors, including rectal cancer, develop immune resistance through diverse mechanisms leading to immune tolerance. PD-1/PD-L1 emerges as the pivotal immune checkpoint pathway in this context (11). PD-1, predominant in activated T-lymphocytes, interacts with PD-L1 on tumor cells, suppressing effector T-cell immune functionality (12). Notably, radiotherapy, especially ionizing radiation, amplifies the anti-tumor immune response of immune checkpoint inhibitors. This amplification is realized by promoting cytotoxic T-cell activity, increasing antigen production, and fostering synergy with immune checkpoint inhibitors (13).

The approach that starts with neoadjuvant chemoradiotherapy followed by sequential combined immunotherapy, primarily as consolidation therapy, was first reported by the investigators of the Japanese VOLTAGE-A study. This study used a conventional long course of radiotherapy with capecitabine and sequential ravulizumab immunotherapy for five courses after the end of radiotherapy. They found that 11 of 37 MSS-type patients achieved a PCR rate of 30%, with three reaching near PCR (8%) and one attaining clinical complete remission (CCR), leading to watchful waiting. Additionally, three of five MSI-H–type patients realized a PCR rate of 60% (14). In another study by Shamseddine et al., a combination of short-term radiotherapy mFOLFOX-6 and avelumab treated locally advanced rectal adenocarcinoma. This study enrolled 13 MSS-type patients, of which three (25%) achieved PCR (tumor regression grade 0), three (25%) approached PCR (tumor regression grade 1), and six (50%) manifested a major pathologic response (15). Recent clinical trials combining neoadjuvant chemoradiotherapy with immunotherapy for LARC have predominantly commenced in the last 2 years. Most are phase II clinical trials focusing on PCR rate as the primary endpoint. Preliminary results from several immunoclinical trials confirm that an improved PCR rate is vital for anus preservation.

Although the efficacy of neoadjuvant radiotherapy combined with immunotherapy is being widely investigated, data on its impact on advanced MSS rectal cancer remain sparse due to limited sample sizes. Thus, this study systematically evaluates the efficacy of neoadjuvant chemoradiotherapy coupled with immunotherapy in MSS/pMMR-type patients with LARC, aiming to offer renewed clinical guidance.

## 2 Methods

### 2.1 Literature search strategy

Two researchers independently executed a detailed and systematic exploration of databases, including PubMed, Embase, Cochrane Library, Web of Knowledge, and ClinicalTrials.gov, China National Knowledge Infrastructure (CNKI), along with other sources, such as conference papers (e.g., https://ascopubs.org/doi/10.1200). The following search terms were used: rectal cancer, *nivolumab*, *camrelizumab*, *sintilimab*, *tislelizumab*, *pembrolizumab*, *toripalimab*, *durvalumab*, *avelumab*, *atezolizumab*, *PD1/PD-L1*, *neoadjuvant*, *preoperative avelumab*, and *atezolizumab*. Logical operators (AND/OR) facilitated combining subject terms with free words. The search strategy adhered to the PRISMA (Preferred Reporting Items for Systematic Reviews and Meta-Analyses) statement (16).

### 2.2 Inclusion and exclusion criteria

For inclusion, the articles considered had to meet the following criteria: the study population should have comprised patients with advanced rectal cancer, specifically those with the MSS/pMMR type. The intervention under investigation must have been neoadjuvant chemoradiotherapy combined with immunotherapy for LARC. Additionally, eligible articles reported on randomized controlled studies, prospective or retrospective studies, or single-arm clinical studies. On the other hand, the exclusion criteria encompassed reviews, commentaries, and other literature types. Studies that were published multiple times, had incomplete data, or from which data could not be extracted were also excluded.

### 2.3 Outcomes

The primary endpoints were PCR (pathologic complete remission), sphincter preservation rate, major pathologic remission (MPR), R0 resection rate (R0 resection represents complete resection of the tumor and negative microscopic margins), and CCR (clinical complete remission).

### 2.4 Data extraction and literature quality evaluation

Two investigators independently reviewed the complete texts, extracted relevant data, and verified the extracted information. Disagreements between them were resolved by a third investigator. Both researchers also assessed the bias risk of the included studies and evaluated the literature quality. If consensus could not be reached, a third researcher intervened to assess the quality of the literature in question. The Newcastle/Ottawa Scale (NOS) was employed to gauge the quality of controlled and single-arm trials (17).

### 2.5 Statistical analysis

Stata 12.0 (StataCorp, College Station, TX, USA) software was used for data processing, and there was significant heterogeneity in the included literature, so the heterogeneity analysis was integrated using a random effects model. Additionally, publication bias was detected using Begg’s test, Egger’s test, and funnel plot analysis.

## 3 Results

### 3.1 Literature screening and quality evaluation

**Figure 1:**
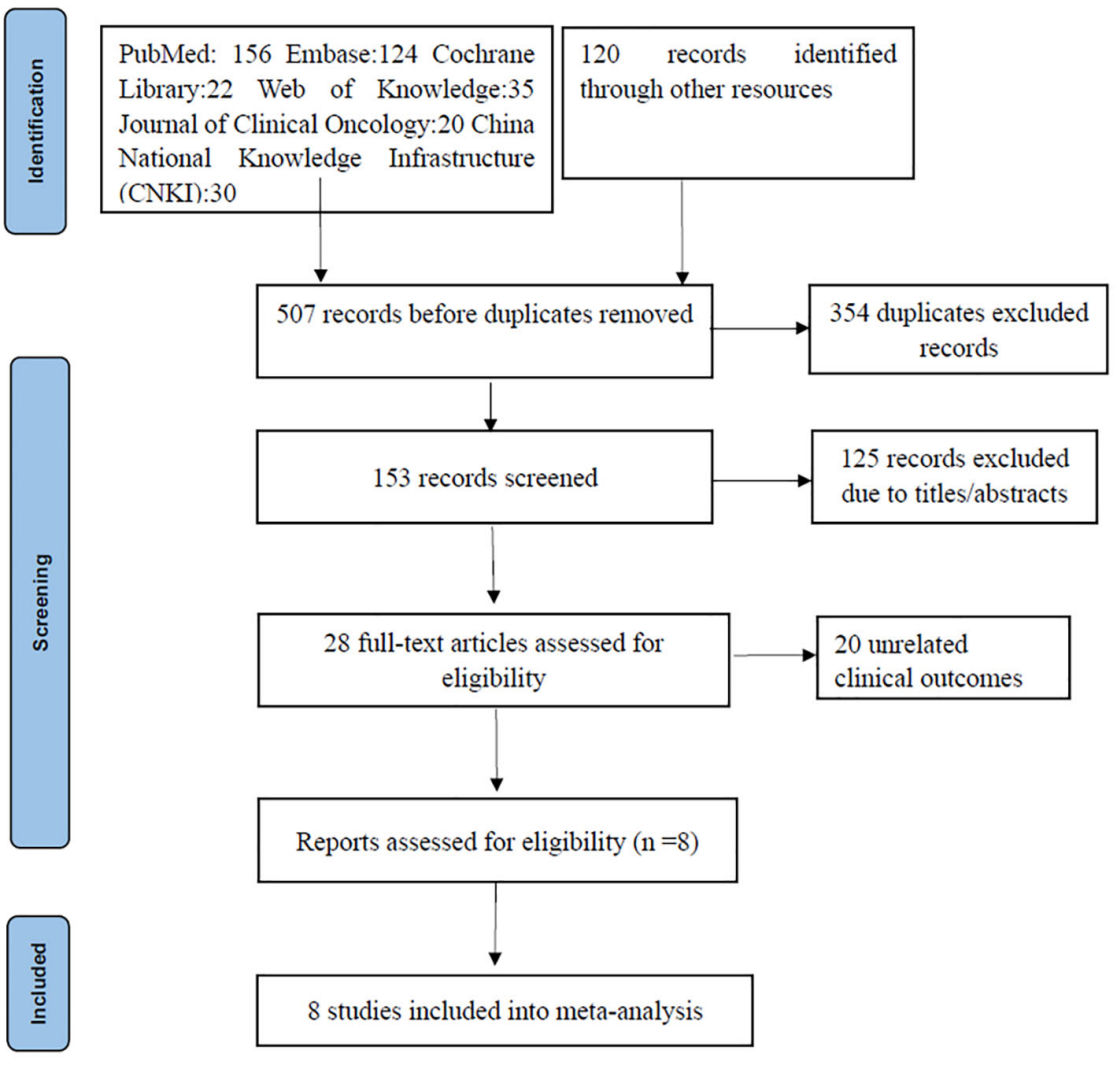
Flow chart of study selection PRISMA.

A thorough search of the literature database yielded 507 articles. After duplicates were removed using Endnote, 153 articles remained. Subsequent screening of titles and abstracts narrowed the pool down to 28 articles. Of these, 20 were excluded due to them being review or commentary articles or not being relevant to the predefined study indicators. Ultimately, eight articles were selected for inclusion, encompassing 204 MSS-type LARC patients who underwent neoadjuvant chemoradiotherapy coupled with immunotherapy (14, 15, 18–23) (**Figure 1**). Out of the included studies, five case-control studies achieved an NOS score of 8, while the other three single-arm studies each scored 6.

### 3.2 Baseline information of the included studies

Key details from the incorporated studies, such as the research program, country, sample size, patients’ pathologic characteristics, study design, study outcomes, and NOS scores, are summarized in **Tables 1**, **2**.

**Table 1:** Baseline information of included studies.

Study	country	Sample Size	Patient characteristics	Clinical Trial Registration Number	Treatment Programs	NOS score
George 2019 (18)	American	45	Stage II/III; MSS	NCT03102047	CRT[Capeox + 50.4 Gy] +Durvalumab × 4 - TME	6
Shamseddine 2020 (15)	Belgium	44	stage II/III;MSS	NCT03503630	5×5Gy+mFOLFOX × 6 + Ave × 6 -TME	8
Lin 2021 (19)	China	30	T3-4N0M0 or T1-4N+M0;MSS	NCT04231552	5 × 5Gy + Capeox × 2 + Camrelizumab× 2 - TME	8
Li 2021 (22)	China	24	pMMR/MSS	NCT02864849	(sintilimab+Capeox) ×3+IMRT+ Capeox×2-TME	8
Bando 2022 (14)	Japan	39	cT3-4N0-2M0;III; MSS	NCT02948348	CRT [Capeox + 50.4 Gy] +Nivolumab × 5- TME	8
Zhou 2022 (20)	China	23	T1-3aN0-1M0; pMMR/MSS	NCT05215379	CRT [Capeox + 50.4 Gy] + Sintilimab × 2 - Cape/Capeox × 6 + Sintilimab × 2 - TME	6
WU 2022 (21)	China	25	pMMR/MSS	NCT04340401	Capeox×3+ Camrelizumab × 3 - CRT [Capeox + 50.4 Gy] - Capeox × 2 - TME	6
Wang 2023 (23)	China	32*	pMMR/MSS	NCT045182820	consolidation group: 25 Gy/5 Fx+ (CAPOX+ toripalimab) ×6-TME Induction group: (CAPOX+ toripalimab) ×2 + 25 Gy/5 Fx+(CAPOX+PD-1) ×4-TME	8

CRT, chemoradiotherapy; TME, total mesorectal excision; mFOLFOX, modify oxaliplatin + leucovorin + 5-fluorouracil; Capeox, capecitabine + oxaliplatin; mFOLFIRINOX, modify oxaliplatin + irinotecan + calcium folinate + 5-fluorouracil; Cape, capecitabin; 5FU, 5-fluorouracil; AF, LV, leucovorin; OXA, oxaliplatin; FOLFOX, oxaliplatin + calcium folinate + 5-fluorouracil; cCR, clinical complete response; pCR, pathologic complete response; MPR, major pathological response; NAR, neoadjuvant rectal score; HR, Hazard Ratio.*: The subjects included in this study were patients who underwent TME after neoadjuvant chemoradiotherapy combined with immunotherapy, so the sample size included in this article was patients who underwent surgery.

**Table 2:** Outcome indicators for inclusion in the study.

Study	PCR rate	Sphincter preservation rate	MPR rate	R0 resection rate	CCR rate
George 2019 (18)	22.2%	71.1%			31.1%
Shamseddine 2020 (15)	25%	–	50%		
Lin 2021 (19)	48.1%	88.9%		100%	
Li 2021 (22)	30%	80%	20%	95%	13.6%
Bando 2022 (14)	29.7%	87.1%		70.3%	
Zhou 2022 (20)	20%	95.5			43.4%
WU 2022 (21)	33.3%	–			
Wang 2023 (23)	56.3%	100%			34.4%

pCR, pathologic complete response; MPR, major pathological response;”-”, not reported.

### 3.3 Study outcomes

#### 3.3.1 PCR rate

**Figure 2:**
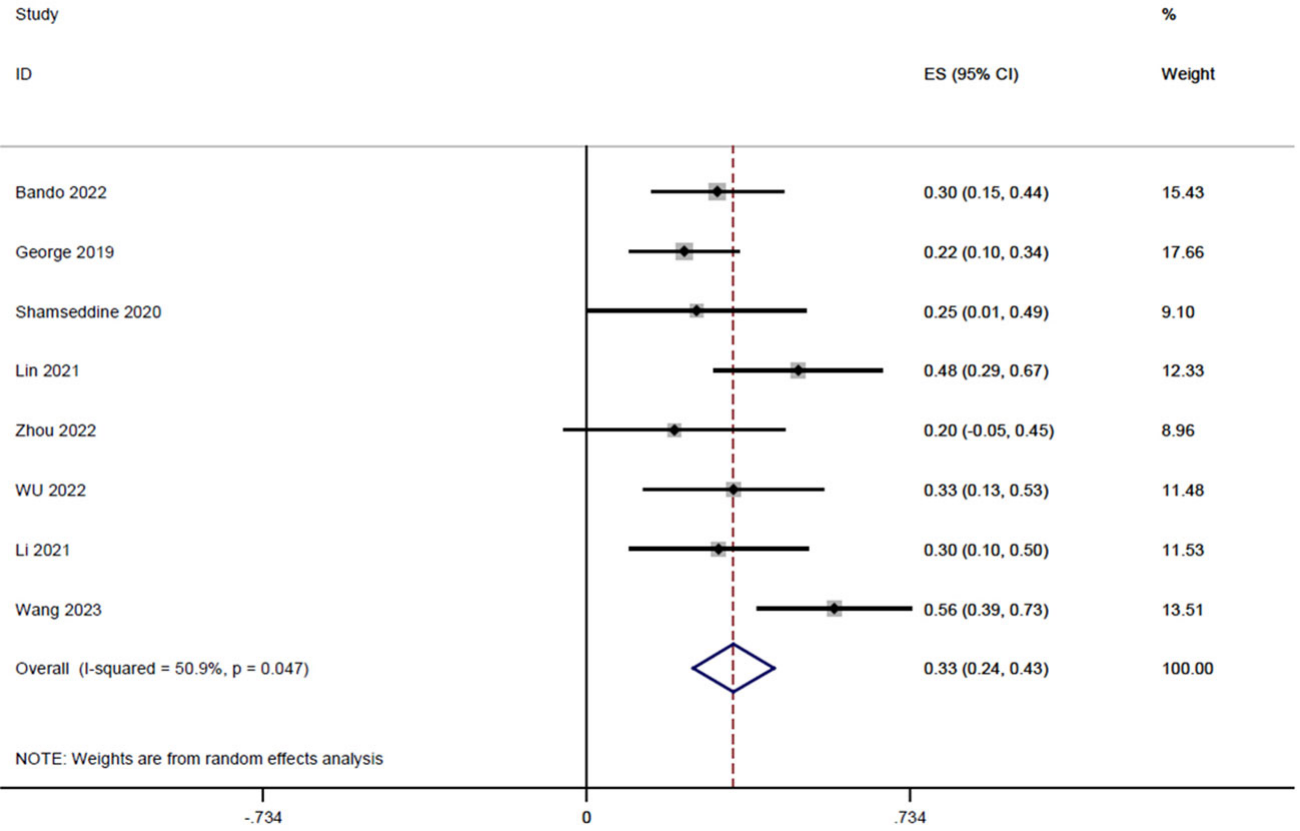
Forest plot of single arm integrated analysis of PCR rate in rectal cancer patients with MSS type.

Eight studies (14, 15, 18–23), with a total of 204 patients, reported PCR rates in a range of 0.2-0.56, and the result of the integrated analysis of PCR rates in this study was 0.33 (95% CI: 0.24-0.43, I^2 = ^50.9%; **Figure 2**).

#### 3.3.2 Sphincter preservation rate

**Figure 3:**
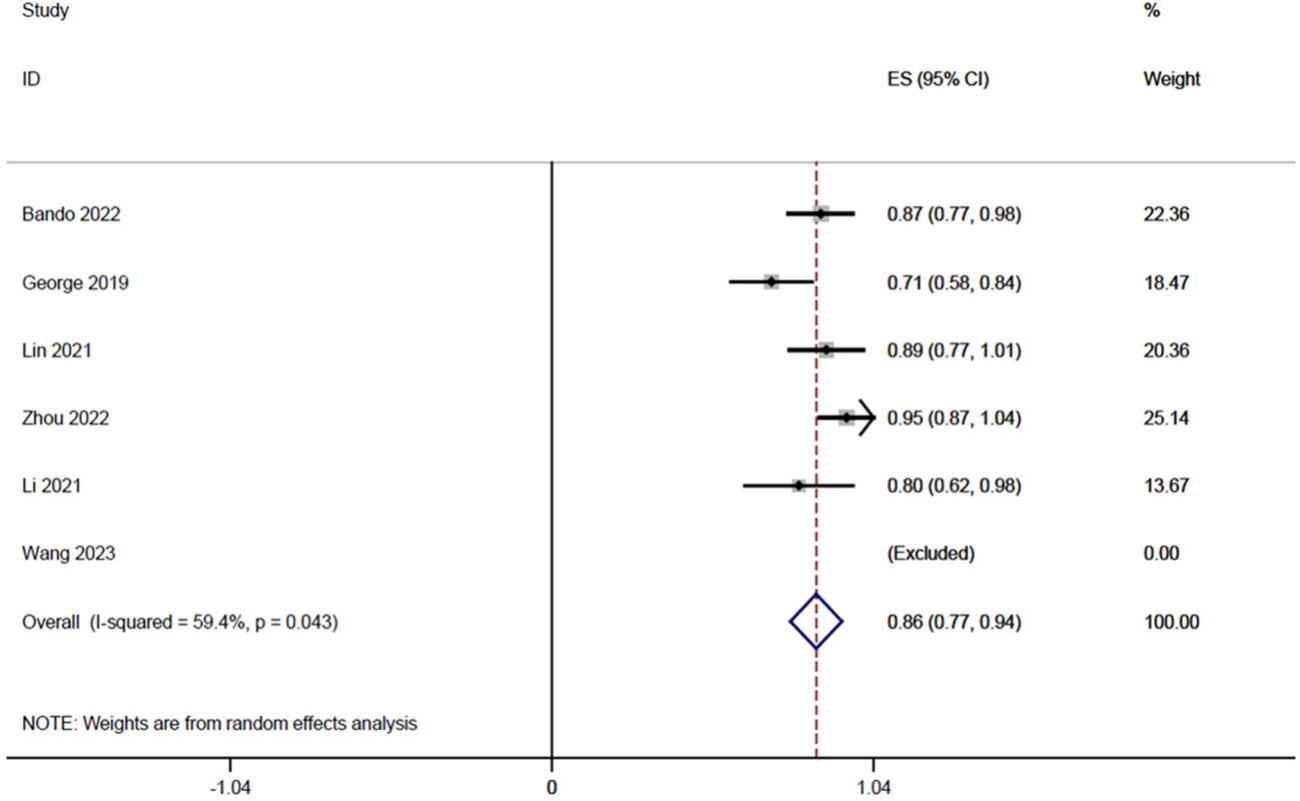
Forest plot of single-arm integrated analysis of mean sphincter preservation rate in patients with MSS-type rectal cancer.

Six studies (14, 18–20, 22, 23), totaling 185 patients, reported sphincter retention rates in a range of 0.71-1, and the integrated analysis of sphincter retention in this study was 0.86 (95% CI: 0.77-0.94, I^2 = ^59.4%; **Figure 3**).

#### 3.3.3 MPR rate

**Figure 4:**
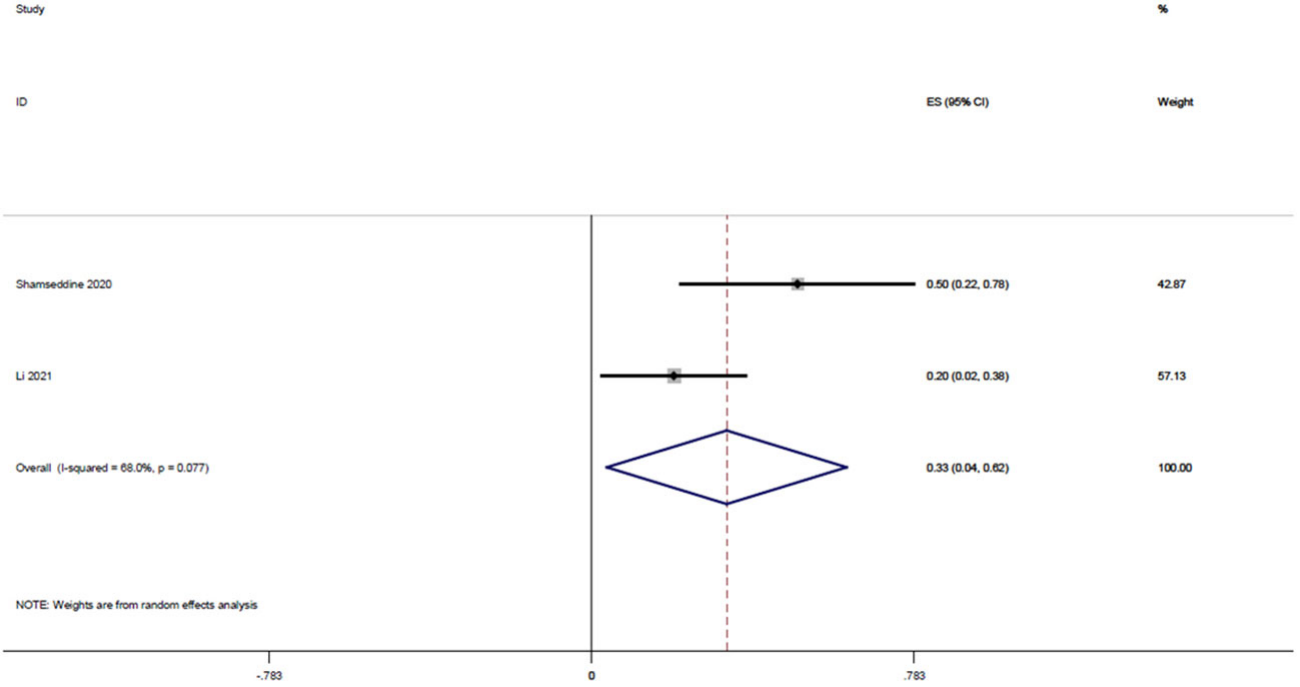
Forest plot of single arm integrated analysis of MPR rate in rectal cancer patients with MSS type.

Two studies (15, 22), with a total of 32 patients, reported MPR rates of 0.2 and 0.5, respectively, and the result of the integrated analysis of MPR rates in this study was 0.33 (95% CI: 0.04-0.62, I^2 = ^68.0%; **Figure 4**).

#### 3.3.4 R0 resection rate

**Figure 5:**
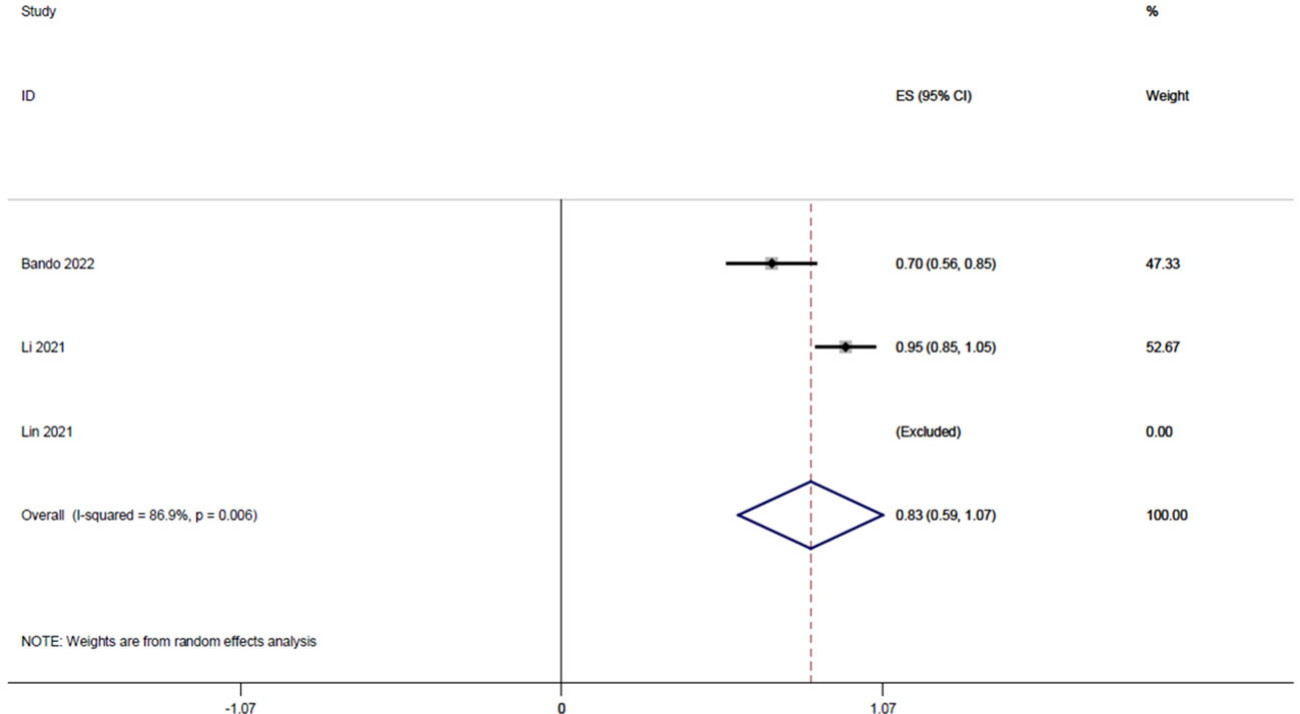
Forest plot of single-arm integrated analysis of R0 resection rate in patients with MSS type of rectal cancer.

Three studies (14, 19, 22), with a total of 84 patients, reported R0 resection rates of 0.71, 1, and 0.95, respectively, and the integrated analysis of R0 resection rates in this study resulted in 0.83 (95% CI: 0.59-1.07, I^2 = ^86.9%; **Figure 5**).

#### 3.3.5 CCR rate

**Figure 6:**
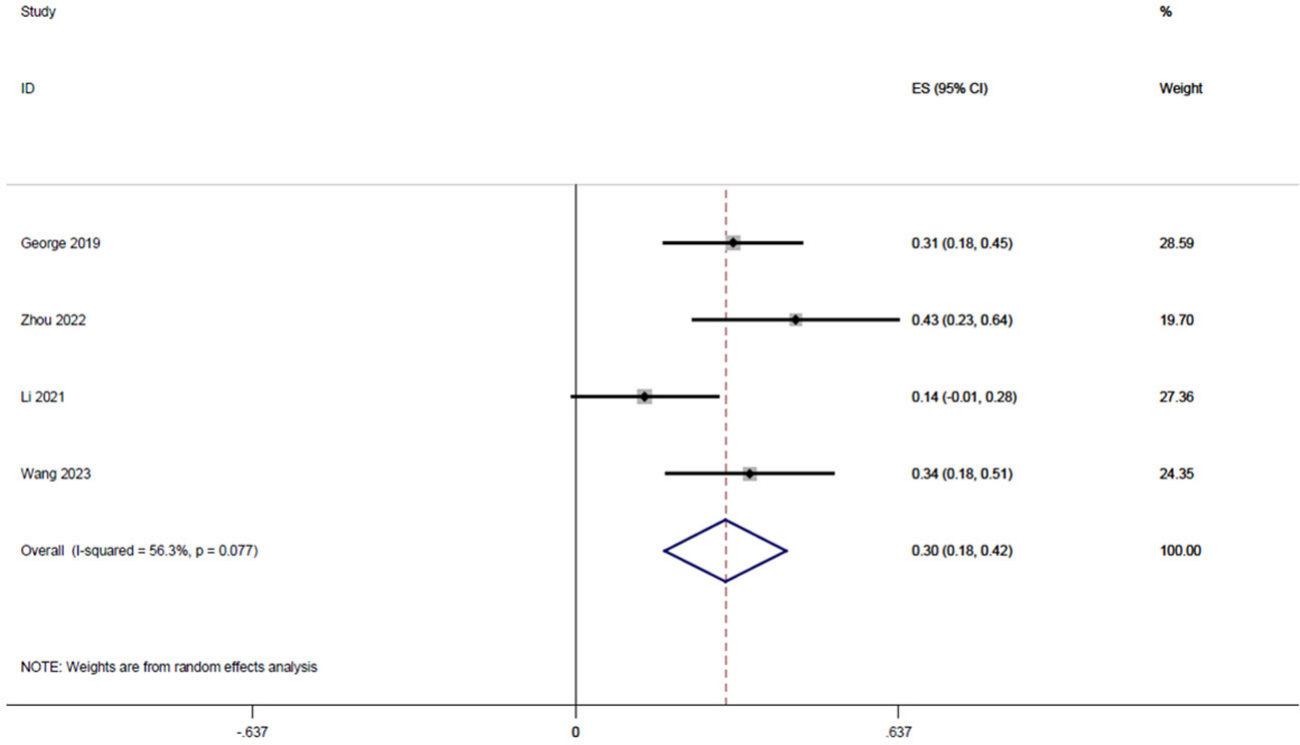
Forest plot of single arm integrated analysis of CCR rate in rectal cancer patients with MSS type.

Four studies (18, 20, 22, 23), with a total of 122 patients, reported CCR rates of 0.31, 0.43, 0.13, and 0.34 respectively, and the integrated analysis of CCR rates in this study resulted in 0.30 (95% CI: 0.18-0.42, I^2 = ^56.3%; **Figure 6**).

## 4 Publication bias

**Figure 7:**
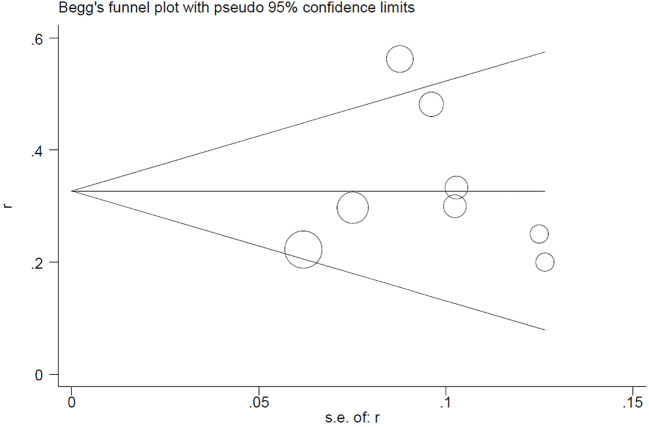
Begg’s funnel plot for PCR rate.

**Figure 8:**
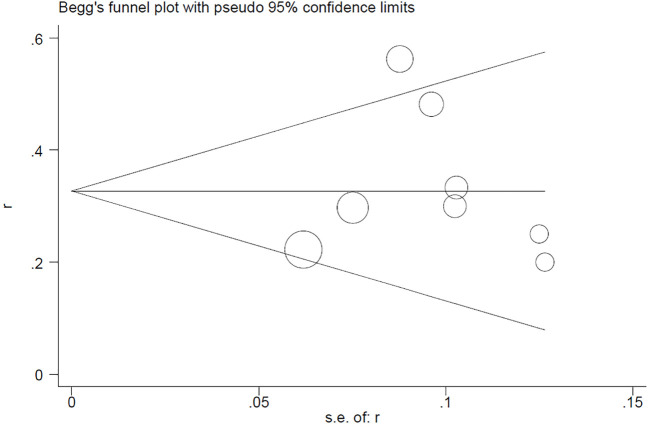
Egger’s funnel plot for PCR rate.

Begg’s test for the PCR rate yielded Pr>|z| = 0.276 (**Figure 7**). Meanwhile, Egger’s test showed Pr>|t| = 0.183 (95% CI: -0.25-0.75; **Figure 8**), indicating no observed publication bias.

## 5 Discussion

Innovations in neoadjuvant therapy for LARC have steadily progressed. According to the NCCN guidelines, long-range radiotherapy (50 Gy/25 Fx) combined with 5-FU or capecitabine concurrently and short-range radiotherapy (25 Gy/5 Fx), which markedly reduces local recurrence rates, are now considered standard-of-care recommendations (24, 25). Nonetheless, the PCR rate of neoadjuvant therapy stands at a mere 10-20%, leaving much to be desired regarding long-term prognosis.

However, immunotherapy has emerged as a revolutionary treatment for various malignant tumors. Preclinical studies have shown that radiotherapy can bolster anti-tumor immune responses. It can also cause an upsurge in PD-L1 expression in tumor tissues, heightening their susceptibility to immunotherapy. Additionally, when combined with immune checkpoint inhibitors, radiotherapy can modulate the tumor microenvironment, mitigating its immunosuppressive effects (26, 27). Furthermore, clinical research has unveiled a “distant effect” wherein chemoradiotherapy paired with immunotherapy leads to significant regression in both the irradiated tumor component and distant tumor tissue, a phenomenon attributed to the systemic immune response triggered by radiotherapy (28, 29).

This synergy offers promising avenues in the neoadjuvant treatment of LARC. Combining neoadjuvant radiotherapy and immunotherapy could potentially break through the bottleneck slowing efforts to improve outcome data related to PCR and CCR in MSS rectal cancer responses. Clinical trials exploring this combination have been steadily progressing, yielding positive results in terms of enhanced tumor regression and improved PCR rates in both MSI-H and MSS rectal cancer patients. This therapeutic approach also increases the probability of anal preservation, bolstering the feasibility of a “wait-and-see” strategy.

In our investigation, we evaluated the efficacy of neoadjuvant chemoradiotherapy in tandem with immunotherapy for patients diagnosed with MSS-type rectal cancer. We incorporated seven clinical trials, encompassing a total of 172 MSS-type rectal cancer patients. An aggregated analysis revealed a PCR rate of 29% after treatment, a substantial improvement compared with using neoadjuvant chemoradiotherapy in isolation. At the 2020 ASCO meeting, findings from the Japanese VOLTAGE -A study were presented. They applied a standard long course of radiotherapy combined with capecitabine, followed by five rounds of sequential ravulizumab immunotherapy after radiotherapy. Out of 37 MSS-type patients, 11 achieved PCR (30%), three achieved near PCR (8%), and one reached CCR, opting for a watchful waiting approach. Given that traditional radiotherapy combined with capecitabine typically reaches a PCR rate of 15-20%, these results (30% PCR) indicate superior efficacy when paired with immunotherapy (14). The recently released NRG-GI002 study showcased the pembrolizumab cohort study results. All cohorts followed the TNT (total neoadjuvant therapy) model, involving eight cycles of FOLFOX (oxaliplatin + folinic acid + 5-fluorouracil) chemotherapy, with the control group receiving sequential long-term radiotherapy (alongside capecitabine). In contrast, the experimental group had the same but combined with pembrolizumab. The PCR rate was 29.4% for the control group and 31.9% for the experimental group, underlining the effectiveness of integrating neoadjuvant chemoradiotherapy with immunotherapy (30). A phase II clinical trial, known as the averectalstudy, was conducted in Lebanon and Jordan. It combined short-course radiotherapy with mFOLFOX6 (oxaliplatin + folinic acid + 5-fluorouracil adjusted regimen 6) chemotherapy and avelumab immunotherapy. Of the 44 patients enrolled, four were excluded for various reasons. Among the remaining 40, 15 achieved PCR (37.5%), and 12 (30%) achieved near PCR with a tumor regression grade of 1. This means that 67.5% of the patients demonstrated significant tumor regression (15).

In 2004, Prof. Habr-Gama from Brazil introduced the “wait-and-see” strategy for patients achieving CCR after neoadjuvant therapy for rectal cancer (nCRT). This approach has shown marked improvements in patient quality of life without impacting long-term survival (31). However, after conventional nCRT, the CCR rates remain less than optimal. A study by Martens et al. showed that out of 141 rectal cancer patients treated with nCRT, only 24 (17%) achieved CCR (32).

Furthermore, a recent evaluation at the Cancer Hospital of Peking University in China assessed the outcomes of the PD1 antibody combined with full neoadjuvant chemoradiotherapy for high-risk, locally progressive, low- and intermediate-stage rectal cancer patients. Among the 24 MSS-type rectal cancer patients, 19 had R0 resection, 16 underwent anal sphincter preservation surgery, and the PCR rate stood at 30.0% (6 out of 20). An additional 20.0% (4 out of 20) had major pathologic responses. The study showed that the combination of PD-1 antibody with full neoadjuvant chemoradiotherapy yielded positive safety and histopathologic regression outcomes. Combining histologic and genetic testing can further assist in identifying individuals who may benefit most from this approach (22).

Our comprehensive analysis of neoadjuvant chemoradiotherapy combined with immunotherapy yielded the following findings associated with this strategy: a CCR rate of 28%, an R0 resection rate of 83%, and an anal preservation rate of 86%. These findings underscore the potential of this combination to enhance the regression of MSS-type rectal tumors, elevate the PCR rate, and offer a safe and tolerable treatment option. It emerges as a viable choice for those patients keen on organ preservation and achieving CCR. This combination serves as a promising option for LARC patients opting for the “wait-and-see” approach and desiring CCR.

Our study has integrated and analyzed the most recent clinical efficacy data regarding neoadjuvant chemoradiotherapy paired with immunotherapy for MSS-type advanced colorectal cancer. The findings solidify the potential clinical significance of this combined approach for MSS-type advanced colorectal cancer patients. The synergy of nCRT and immunotherapy for treating LARC leads to favorable PCR/CCR rates and increases the probability of preserving the anus. It is crucial to note that our study had a limited sample size. Additionally, differences in treatment regimens and the sequence of applying radiotherapy and immunotherapy across studies led to heterogeneity in outcomes. Nonetheless, in the evolving landscape of immunotherapy, while radiotherapy remains pivotal in treating rectal cancer, we are optimistic that immunotherapy combined with neoadjuvant chemoradiotherapy heralds a brighter future for patients with the MSS type.

## 6 Conclusion

Our comprehensive analysis, encompassing eight single-arm clinical studies, underscores the promising efficacy of neoadjuvant chemoradiotherapy in tandem with immunotherapy for patients with MSS-type LARC. This combined approach has demonstrated notable enhancements in outcomes, including the PCR rate, MPR rate, R0 resection rate, major sphincter preservation rate, and CCR rate. Given these promising preliminary findings, the combination of neoadjuvant chemoradiotherapy with immunotherapy heralds vast potential in the therapeutic landscape. Nonetheless, for a definitive endorsement of its efficacy, there is a pressing need for large-scale, randomized controlled trials focusing on MSS-type LARC.

## Data availability statement

The original contributions presented in the study are included in the article/**Supplementary Material**. Further inquiries can be directed to the corresponding author.

## Author contributions

YY: Data curation, Writing – original draft. MC: Conceptualization, Data curation, Formal Analysis, Writing – review & editing. XX: Conceptualization, Investigation, Software, Writing – review & editing. MW: Conceptualization, Investigation, Supervision, Validation, Writing – review & editing. QW: Writing – review & editing. BZ: Formal Analysis, Methodology, Validation, Writing – review & editing.
